# Supplementary material for: Alkyl Chain Engineering of Low Bandgap Non-Fullerene Acceptors for High-Performance Organic Solar Cells: Branched vs. Linear Alkyl Side Chains
Source: Polymers (Basel). 2022 Sep 12;14(18):3812. doi: 10.3390/polym14183812 (PMC9502987; doi:10.3390/polym14183812)
Supplement: Supplementary file 1 [file polymers-14-03812-s001.zip › polymers-1816111-supplementary.pdf]

polymers

MDPI

Supplementary Material

Alkyl Chain Engineering of Low Bandgap Non-Fullerene Acceptors for High-Performance Organic Solar Cells: Branched vs. Linear Alkyl Side Chains

Youngwan Lee [1,†], Telugu Bhim Raju [1,†], Hyerim Yeom [2,†], Peddaboodi Gopikrishna [1], Kwangmin Kim [1], Hye Won Cho [2], Jung Woo Moon [3], Jeong Ho Cho [3], Jin Young Kim [2,5,*] and BongSoo Kim [1,4,5,*]

[1] Department of Chemistry, Ulsan National Institute of Science and Technology (UNIST), 50 UNIST-gil, Ulsan 44919, Korea
[2] School of Energy and Chemical Engineering, Ulsan National Institute of Science and Technology (UNIST), 50 UNIST-gil, Ulsan 44919, Republic of Korea
[3] Department of Chemical and Biomolecular Engineering, Yonsei University, Seoul 03722, Korea
[4] Graduate School of Semiconductor Materials and Device Engineering, Ulsan National Institute of Science and Technology (UNIST), 50 UNIST-gil, Ulsan 44919, Korea
[5] Graduate School of Carbon Neutrality, Ulsan National Institute of Science and Technology (UNIST), 50 UNIST-gil, Ulsan 44919, Korea
* Correspondence: jykim@unist.ac.kr (J.Y.K.); bongsoo@unist.ac.kr (B.K.); Tel.: +82-52-217-2911 (J.Y.K.); +82-52-317-3197 (B.K.); Fax: +82-52-217-2909 (J.Y.K.); +82-52-317-2297 (B.K.)
† These authors contributed equally to this work.

EXPERIMENTAL SECTION

Materials and Methods

All reagents were purchased from the commercial sources and directly used unless otherwise noted. IEHICO-4F was purchased from 1-Material company and solvents were obtained from Daejung. All solvents used without further purification except THF that was dried over sodium and benzophenone before being distilled. All reactions were performed under the argon atmosphere in glasswares that were pre-dried in an oven, unless otherwise noted.

^{1}H and ^{13}C-NMR spectra were obtained to identify the molecular structures of all the synthesized compounds on a Bruker Advance III 400MHz using $CDCl_3$ as a solvent. UV/vis absorption spectroscopy was performed on a Cary 300 UV-vis spectrophotometer (Agilent technologies). The Solution-state UV-Vis absorbance of the IRICO-4F was measured at a concentration of 0.01 mg/mL which is dissolved in chloroform. Each NFA was dissolved in chlorobenzene with a concentration of 20 mg/mL and the solutions were spin-coated on the bare glass to measure the film state UV-Vis absorbance. The molecular mass of the synthesized were confirmed by using an Ultraflex III matrix assisted laser desorption ionization mass spectrometer (MALDI-TOF). Cyclic voltammetry (CV) was conducted using an Electrochemical Workstation CS120 (CorrTest instruments). A 0.1 M tetrabutylammonium hexafluorophosphate ($TBAPF_6$) solution in anhydrous

CH_3CN was used as an electrolyte. A standard three-electrode cell was chosen for this study. Pt wires were used as working and counter electrodes, and a silver wire for pseudo reference electrode. All the measurements were done under the N_2 atmosphere. After thorough purging N_2 gas inside CV solution, voltage was scanned at the rate of 100 mV/s. HOMO and LUMO levels were determined from the onsets values of the oxidation and reduction curves, respectively. Differential scanning calorimetry (DSC) was run on a TA Instrument Q200.

Grazing-incidence wide-angle X-ray scattering (GIWAXS) measurements were performed at the 6D beam line of the Pohang Accelerator Laboratory in Republic of Korea. The energy of the X-ray was 11.564 keV and the incidence angle was 0.11° with 100 s of the X-ray irradiation time. GIWAXS patterns were recorded by using a 2D-CCD detector (MX225-HS, Rayonix LLC, USA). Atomic force microscopy (AFM) measurement was carried out on a Veeco dimension AFM. in a tapping mode at ambient conditions. All the blend films and neat films were spin coated on ITO substrates. Transmission electron microscopy (TEM) images of the films were acquired by using a JEOL JEM-1400 transmission electron microscope operated at 80 kV. The specimens for TEM investigation were prepared by spin coating the solutions on PEDOT:PSS substrates, then floating the films on a water surface, and transferring to TEM grids.

Synthetic Procedures.

Synthesis of 3-Methoxythiophene (2). A solution of 30 wt % sodium methoxide in methanol (1.8 mL, 98.14 mmol, 1.6 equiv) was added to a two-neck round-bottom flask under the Ar. Then, 3-bromothiophene (1.00 g, 61.33 mmol) and CuBr (0.087 g, 6.13 mmol, 0.1 equiv) were added successively with stirring and the mixture was allowed to stir at 65 °C for 16 h. After cooling, the reaction mixture was poured into water (300 mL) and extracted three times with diethyl ether (100 mL). The ether solution was dried with anhydrous $MgSO_4$ and then concentrated under reduced pressure. Afforded the product as a colorless liquid (0.5 g, 50%). ^{1}H-NMR (400 MHz, $CDCl_3$): δ 7.18 (dd, 1H), 6.76 (dd, 1H), 6.25 (dd, 1.01H), 3.82 (s, 3.76H).

Synthesis of 3-(octyloxy)thiophene (3a). A two-neck flask was equipped with a condenser, which connected to a dry N_2. To this flask, 10 mL of toluene was added, followed by the sequential addition of 3-methoxythiophene (0.35 g, 3.06 mmol), 1-octanol (0.8 mL, 20.8 mmol, 2.0 equiv), and *p*-toluenesulfonic acid monohydrate (0.02 g, 0.30 mmol, 0.1 equiv). The mixture was heated to 120 °C under N_2 for 20 h. After cooling, the mixture was poured into 150 mL of water and extracted three times with DCM. The organic layer was washed with water twice and then dried over $MgSO_4$. After

solvent evaporation under reduced pressure, the residue was purified by column chromatography with hexane the product was obtained as a colorless liquid (0.33 g, 60%). ^{1}H NMR (400 MHz, $CDCl_3$): *δ* 7.18 (dd, *J* = 5.2, 3.1 Hz, 1H), 6.78 (dd, *J* = 5.2, 1.5 Hz, 1H), 6.24 (dd, *J* = 3.1, 1.6 Hz, 1H), 3.96 (t, *J* = 6.6 Hz, 2H), 1.84 – 1.76 (m, 2H), 1.47 (dd, *J* = 9.9, 5.1 Hz, 2H), 1.35 – 1.31 (m, 8H), 0.93 – 0.91 (m, 3H). ^{13}C NMR (101 MHz, $CDCl_3$): *δ* 158.29, 124.65, 119.73, 97.10, 70.45, 32.06, 31.84, 29.61, 29.52, 29.48, 26.30, 22.90, 14.32.

Synthesis of 3-(decyloxy)thiophene (3b). Followed by the above procedure 3a

^{1}H NMR (400 MHz, $CDCl_3$): *δ* 7.17 (dd, *J* = 5.3, 3.1 Hz, 1H), 6.77 (dd, *J* = 5.2, 1.5 Hz, 1H), 6.24 (dd, *J* = 3.2, 1.6 Hz, 1H), 3.96 (t, *J* = 6.5 Hz, 2H), 1.79 (dt, *J* = 8.3, 6.6 Hz, 2H), 1.47 (dd, *J* = 9.9, 5.9 Hz, 2H), 1.33 – 1.30 (m, 12H), 0.92 (s, 3H). ^{13}C NMR (101 MHz, $CDCl_3$): *δ* 158.30, 124.64, 119.73, 97.09, 70.45, 32.16, 31.86, 29.83, 29.66, 29.59, 29.54, 26.32, 22.91, 14.32.

Synthesis of 3-(2-butylethyloxy)thiophene (3e). Followed by the above procedure 3a.

^{1}H NMR (400 MHz, Chloroform-d): *δ* 7.17 (dd, J = 5.3, 3.2 Hz, 1H), 6.76 (dd, J = 5.3, 1.6 Hz, 1H), 6.23 (dd, J = 3.1, 1.5 Hz, 1H), 3.84 (d, J = 5.8 Hz, 2H), 1.69 – 1.62 (m, 1H), 1.46 (td, J = 7.6, 6.4 Hz, 5H), 0.93 (t, J = 7.4 Hz, 7H). ^{13}C NMR (101 MHz, Chloroform-d): *δ* 158.53, 124.64, 119.84, 96.99, 72.61, 41.15, 23.58, 11.36.

Synthesis of 2-bromo-3-(octyloxy)thiophene (4a). *N*-bromosuccinimide (0.30 g, 1.73 mmol, 1.0 equiv) was added portion wise into 3-(octyloxy)thiophene (0.32 g, 1.73 mmol) in 20 mL of DCM at 0 °C. The mixture was warmed to room temperature and stirred for 2 h. After completion of the reaction, it was poured into 200 mL of water followed by extraction with 100 mL of chloroform. The organic layer washed with water twice and then dried over $MgSO_4$ before being concentrated under reduced pressure. The crude product was purified by column chromatography with hexane as the eluent and the product was obtained as a yellowish liquid (0.29 g, 65%). ^{1}H NMR (400 MHz, $CDCl_3$): *δ* 7.18 (d, *J* = 5.9 Hz, 1H), 6.74 (d, *J* = 6.0 Hz, 1H), 4.03 (t, *J* = 6.6 Hz, 2H), 1.79 – 1.72 (m, 2H), 1.48 – 1.42 (m, 2H), 1.32 – 1.27 (m, 8H), 0.89 (s, 3H). ^{13}C NMR (101 MHz, $CDCl_3$): *δ* 154.77, 124.33, 117.75, 91.82, 72.49, 32.02, 31.82, 29.70, 29.52, 29.44, 26.04, 22.88, 14.35, 14.33, 1.24.

Synthesis of 2-bromo-3-(decyloxy)thiophenene (4b). Followed by the above synthetic procedure 4a.

^{1}H NMR (400 MHz, $CDCl_3$) δ 7.18 (d, *J* = 5.9 Hz, 1H), 6.74 (d, *J* = 5.9 Hz, 1H), 4.04 (t, *J* = 6.6 Hz, 2H), 1.77 (dt, *J* = 14.7, 6.7 Hz, 2H), 1.49 (d, *J* = 2.1 Hz, 2H), 1.39 – 1.24 (m, 12H), 0.93 – 0.89 (m, 3H). ^{13}C NMR (101 MHz, $CDCl_3$) δ 154.79, 124.26, 117.69, 91.81, 72.44, 32.15, 31.85, 29.81, 29.80, 29.74, 29.59, 29.57, 26.07, 22.93, 22.90, 14.32.

Synthesis of 2-bromo 3-(2-ethylbutoxy)thiophene (4c). Followed by the above synthetic procedure 4a.

^{1}H NMR (400 MHz, Chloroform-d): δ 7.18 (d, J = 6.2 Hz, 1H), 6.75 (d, J = 5.9 Hz, 1H), 3.93 (d, J = 5.8 Hz, 2H), 1.66 – 1.61 (m, 1H), 1.52 – 1.43 (m, 5H), 0.94 (t, J = 7.5 Hz, 7H). ^{13}C NMR (101 MHz, Chloroform-d): δ 158.53, 124.64, 119.84, 96.99, 72.61, 41.15, 23.58, 11.36.

Synthesis of 5-bromo-4-(octyloxy)thiophene-2-carbaldehyde (5a).

LDA (1.42 M, 3.72 mL) was added dropwise to the solution of 2-bromo-3-(octyloxy)thiophene (0.29 g, 1.10 mmol) in 5 mL of anhydrous THF at -78 °C under an argon atmosphere. The mixture was stirred for 0.5 h at low temperature, and *N*-formylpiperidine (0.27 mL, 2.75 mmol) was added in one portion, after 10 min, the low temperature bath allowed to the room temperature and stirred for 2 h. Then, the mixture was quenched by water and extracted with diethyl ether for three times. The residue was purified by column chromatography with $CHCl_3$:hexane (1:1) as eluent to afford product as a greenish liquid (0.12 g, 40%). ^{1}H NMR (400 MHz, $CDCl_3$): δ 9.70 (s, 1H), 7.36 (s, 1H), 4.08 (t, *J* = 6.5 Hz, 2H), 1.82 – 1.77 (m, 2H), 1.49 – 1.43 (m, 2H), 1.29 (d, *J* = 5.7 Hz, 8H), 0.89 (s, 3H). ^{13}C NMR (101 MHz, $CDCl_3$): δ 181.51, 155.76, 140.29, 123.81, 105.84, 72.75, 32.00, 31.82, 29.46, 26.00, 22.88, 14.34, 14.31.

Synthesis of 5-bromo-4-(decyloxy)thiophene-2-carbaldehyde (5b). Followed by the above synthetic procedure 5a.

^{1}H NMR (400 MHz, $CDCl_3$): δ 9.70 (s, 1H), 7.36 (s, 1H), 4.08 (t, *J* = 6.6 Hz, 2H), 1.82-1.75 (m, 2H), 1.49-1.43 (m, 2H), 1.36-1.25 (m, 12H), 0.90-0.86 (m, 3H). ^{13}C NMR (101 MHz, $CDCl_3$): δ 181.49, 155.74, 140.28, 123.80, 105.80, 72.73, 32.10, 31.80, 29.74, 29.51, 29.49, 29.47, 25.98, 22.89, 22.87, 14.33.

Synthesis of 5-bromo 4-(2-ethylbutoxy)thiophene-2-carbaldehyde (5c). Followed by the above synthetic procedure 5a.

^{1}H NMR (400 MHz, Chloroform-d): δ 9.70 (s, 1H), 7.38 (s, 1H), 3.98 (d, J = 5.8 Hz, 2H), 1.70 – 1.66 (m, 1H), 1.52 – 1.45 (m, 4H), 0.95 (t, J = 7.5 Hz, 6H).

Synthesis of 5,5'-(4,4,9,9-tetrakis(4-hexylphenyl)-4,9-dihydro-*s*-indaceno[1,2-*b*:5,6-*b'*]dithiophene-2,7-diyl)bis(4-(octyloxy)thiophene-2-carbaldehyde) (7a)

To a solution of compound 5a (0.19 g, 0.68 mmol) and (4,4,9,9-tetrakis(4-hexylphenyl)-4,9-dihydro-sindaceno[1,2-b:5,6-b']dithiophene-2,7-diyl)bis(trimethylstannane) (0.4 g, 0.32 mmol) in anhydrous toluene (8 mL) and DMF (1.6 mL), $Pd(PPh_3)_4$ (18 mg, 5% mol) was added under N_2 atmosphere. After being stirred

at 110 °C for 16 h, the solvent was removed with rotary evaporator. The crude product was purified by column chromatography on silica gel, eluting with $CHCl_3$: hexane (3:1) to afford compound 7 (0.25 g, 60 % yield) as a dark-red solid. ^1H NMR (400 MHz, $CDCl_3$): δ 9.73 (s, 2H), 7.43 (d, J = 4.4 Hz, 4H), 7.35 (s, 2H), 7.18 (d, J = 8.3 Hz, 8H), 7.08 (d, J = 8.4 Hz, 8H), 4.15 (t, J = 6.6 Hz, 4H), 2.59 – 2.55 (m, 8H), 1.91 – 1.85 (m, 4H), 1.63 – 1.57 (m, 8H), 1.54 – 1.49 (m, 4H), 1.37 – 1.27 (m, 49H), 0.88 (t, J = 5.8 Hz, 18H). ^{13}C NMR (101 MHz, $CDCl_3$): δ 181.80, 156.40, 153.95, 153.09, 143.30, 141.85, 141.78, 136.78, 135.87, 135.62, 128.59, 128.07, 128.01, 123.84, 121.41, 117.72, 72.41, 63.08, 35.79, 32.00, 31.92, 31.55, 29.61, 29.43, 29.38, 26.18, 22.84, 22.80, 14.30. MS-MALDI $[M]^+$, $C_{90}H_{110}O_4S_4$ Exact Mass: 1382.73, calcd 1382.65

Synthesis of 5,5'-(4,4,9,9-tetrakis(4-hexylphenyl)-4,9-dihydro-s-indaceno[1,2-*b*:5,6-*b'*]dithiophene-2,7-diyl)bis(4-(decyloxy)thiophene-2-carbaldehyde) (7b)

Followed by the above synthetic procedure 7a.

^1H NMR (400 MHz, $CDCl_3$) δ 9.73 (s, 2H), 7.43 (d, J = 5.4 Hz, 4H), 7.35 (s, 2H), 7.18 (d, J = 8.3 Hz, 8H), 7.08 (d, J = 8.4 Hz, 8H), 4.15 (t, J = 6.6 Hz, 4H), 2.59 – 2.54 (m, 8H), 1.88 (dt, J = 14.5, 6.7 Hz, 4H), 1.63 – 1.55 (m, 9H), 1.50 (q, J = 7.5 Hz, 4H), 1.39 – 1.23 (m, 55H), 0.89 – 0.85 (m, 18H). ^{13}C NMR (101 MHz, $CDCl_3$) δ 181.80, 156.42, 153.95, 153.10, 141.85, 141.79, 136.78, 135.86, 135.61, 128.59, 128.08, 128.03, 123.85, 121.45, 117.73, 72.41, 63.09, 35.80, 32.11, 31.93, 31.56, 29.78, 29.74, 29.62, 29.51, 29.48, 29.39, 26.17, 22.89, 22.81, 14.33, 14.30. MS-MALDI $[M]^+$, $C_{94}H_{118}O_4S_4$ Exact Mass: 1438.79, calcd 1438.66

Synthesis of 5,5'-(4,4,9,9-tetrakis(4-hexylphenyl)-4,9-dihydro-*s*-indaceno[1,2-*b*:5,6-*b'*]dithiophene-2,7-diyl)bis(4-(2-ethylbutoxy)thiophene-2-carbaldehyde) (7c)

Followed by the above synthetic procedure 7a.

^1H NMR (400 MHz, Chloroform-d) δ 9.74 (s, 2H), 7.46 (d, J = 3.9 Hz, 4H), 7.39 (s, 2H), 7.20 (d, J = 8.0 Hz, 8H), 7.10 (d, J = 8.0 Hz, 8H), 4.08 (d, J = 5.4 Hz, 4H), 2.59 (t, J = 7.8 Hz, 8H), 1.79 – 1.74 (m, 2H), 1.64 – 1.53 (m, 17H), 1.37 – 1.28 (m, 26H), 0.96 (t, J = 7.4 Hz, 12H), 0.89 (t, J = 6.4 Hz, 13H). ^{13}C NMR (101 MHz, Chloroform-d) δ 181.74, 156.55, 153.90, 153.31, 142.89, 141.82, 141.76, 136.88, 135.77, 135.61, 128.58, 128.05, 128.00, 127.42, 123.46, 121.55, 117.68, 73.99, 63.04, 41.22, 35.76, 31.90, 31.54, 29.33, 23.56, 22.78, 14.30, 11.32.

Synthesis of 2,2'-((2*Z*,2'*Z*)-(((4,4,9,9-tetrakis(4-hexylphenyl)-4,9-dihydro-*s*-indaceno[1,2-*b*:5,6-*b'*]dithiophene-2,7-diyl)bis(4-(octyloxy)thiophene-5,2-diyl))bis(methaneylylidene))bis(5,6-difluoro-3-oxo-2,3-dihydro-1*H*-indene-2,1-diylidene))dimalononitrile (IOICO-4F)

Compound 7a (0.25 g, 0.18 mmol) and 8 (0.21 g, 0.94 mmol) were added into R.B. flask. After that, chloroform (20 mL) and pyridine (0.2 mL) were added to the reaction mixture under the argon atmosphere. Then, the reaction mixture was placed in an oil bath at 65 °C and stirred for 3 hours. The mixture was directly purified by silica gel column chromatography by using chloroform as the eluent to yield the product as a black solid (0.157 g, yield 75%). ^{1}H NMR (400 MHz, $CDCl_3$) δ 8.68 (s, 2H), 8.50 (dd, *J* = 10.0, 6.5 Hz, 2H), 7.62 (t, *J* = 7.5 Hz, 2H), 7.59 (s, 2H), 7.51 (s, 2H), 7.45 (s, 2H), 7.19 (d, *J* = 8.3 Hz, 8H), 7.11 (d, *J* = 8.4 Hz, 8H), 4.21 (t, *J* = 6.5 Hz, 4H), 2.61 – 2.56 (m, 8H), 1.93 (dd, *J* = 8.5, 6.4 Hz, 4H), 1.64 – 1.58 (m, 8H), 1.55 (s, 8H), 1.43 – 1.26 (m, 52H), 0.89 – 0.86 (m, 18H). ^{13}C NMR (101 MHz, $CDCl_3$) δ 186.30, 158.19, 157.99, 154.98, 154.73, 147.58, 142.16, 141.28, 137.18, 136.45, 136.25, 131.76, 130.13, 128.76, 128.09, 123.74, 120.86, 118.33, 114.72, 72.70, 63.12, 35.81, 32.00, 31.94, 31.80, 31.58, 29.50, 29.41, 29.39, 29.36, 26.19, 22.87, 22.85, 22.81, 14.33. ^{19}F NMR ($CDCl_3$, 400 MHz): δ 123.25, 124.39. MS-MALDI ($[M + H]^+$, $C_{114}H_{114}F_4N_4O_4S_4$ Exact Mass: 1806.77, calcd 1807.53.

Synthesis of 2,2'-((2*Z*,2'*Z*)-(((4,4,9,9-tetrakis(4-hexylphenyl)-4,9-dihydro-*s*-indaceno[1,2-*b*:5,6-*b'*]dithiophene-2,7-diyl)bis(4-(decyloxy)thiophene-5,2-diyl))bis(methaneylylidene))bis(5,6-difluoro-3-oxo-2,3-dihydro-1*H*-indene-2,1-diylidene))dimalononitrile (IDICO-4F)

Followed by the above synthetic procedure IOICO-4F.

^{1}H NMR (400 MHz, $CDCl_3$): *δ* 8.68 (s, 2H), 8.50 (dd, *J* = 10.0, 6.5 Hz, 2H), 7.63 (t, *J* = 7.5 Hz, 2H), 7.59 (s, 2H), 7.51 (s, 2H), 7.45 (s, 2H), 7.19 (d, *J* = 8.3 Hz, 8H), 7.11 (d, *J* = 8.3 Hz, 8H), 4.21 (t, *J* = 6.6 Hz, 4H), 2.61 – 2.56 (m, 8H), 1.98 – 1.90 (m, 4H), 1.60 (q, *J* = 7.5 Hz, 9H), 1.53 (d, *J* = 9.4 Hz, 9H), 1.41 – 1.23 (m, 56H), 0.91 – 0.85 (m, 19H). ^{13}C NMR (101 MHz, $CDCl_3$): *δ* 186.29, 157.99, 154.96, 154.73, 147.56, 142.16, 141.28, 137.18, 136.45, 131.76, 130.13, 128.76, 128.09, 123.76, 120.87, 118.33, 114.76, 72.69, 68.71, 63.12, 35.81, 32.11, 31.94, 31.58, 29.78, 29.75, 29.51, 29.50, 29.45, 29.36, 26.16, 22.89, 22.81, 14.34, 14.31. ^{19}F NMR ($CDCl_3$, 400 MHz): δ 123.22, 124.33. MS-MALDI ($[M + H]^+$, $C_{118}H_{122}F_4N_4O_4S_4$ Exact Mass: 1862.83, calcd 1863.82

Synthesis of 2,2'-((2*Z*,2'*Z*)-(((4,4,9,9-tetrakis(4-hexylphenyl)-4,9-dihydro-*s*-indaceno[1,2-*b*:5,6-*b'*]dithiophene-2,7-diyl)bis(4-(2-ethylbutoxy)thiophene-5,2-diyl))bis(methaneylylidene))bis(5,6-difluoro-3-oxo-2,3-dihydro-1*H*-indene-2,1-diylidene))dimalononitrile (IEBICO-4F)

Followed by the above synthetic procedure IOICO-4F.

^{1}H NMR (400 MHz, Chloroform-d): δ 8.69 (s, 2H), 8.51 (dd, J = 10.0, 6.5 Hz, 2H), 7.65 – 7.60 (m, 4H), 7.49 (d, J = 12.5 Hz, 4H), 7.19 (d, J = 8.3 Hz, 8H), 7.11 (d, J = 8.2 Hz, 8H), 4.12 (d, J = 5.4 Hz, 4H), 2.62 – 2.56 (m, 8H), 1.84 – 1.79 (m, 2H), 1.65 – 1.55 (m, 16H), 1.39 – 1.25 (m, 28H), 0.97 (t, J = 7.5 Hz, 12H), 0.90 – 0.84 (m, 13H). ^{13}C NMR (101 MHz, Chloroform-d): δ 186.39, 158.33, 158.09, 154.94, 147.25, 142.21, 141.27, 137.98, 137.29, 136.56, 136.27, 131.72, 130.01, 128.77, 128.10, 123.87, 118.31, 115.21, 114.76, 74.38, 63.08, 41.22, 35.81, 31.95, 31.82, 31.59, 29.35, 23.64, 22.88, 22.82, 14.35, 11.34.

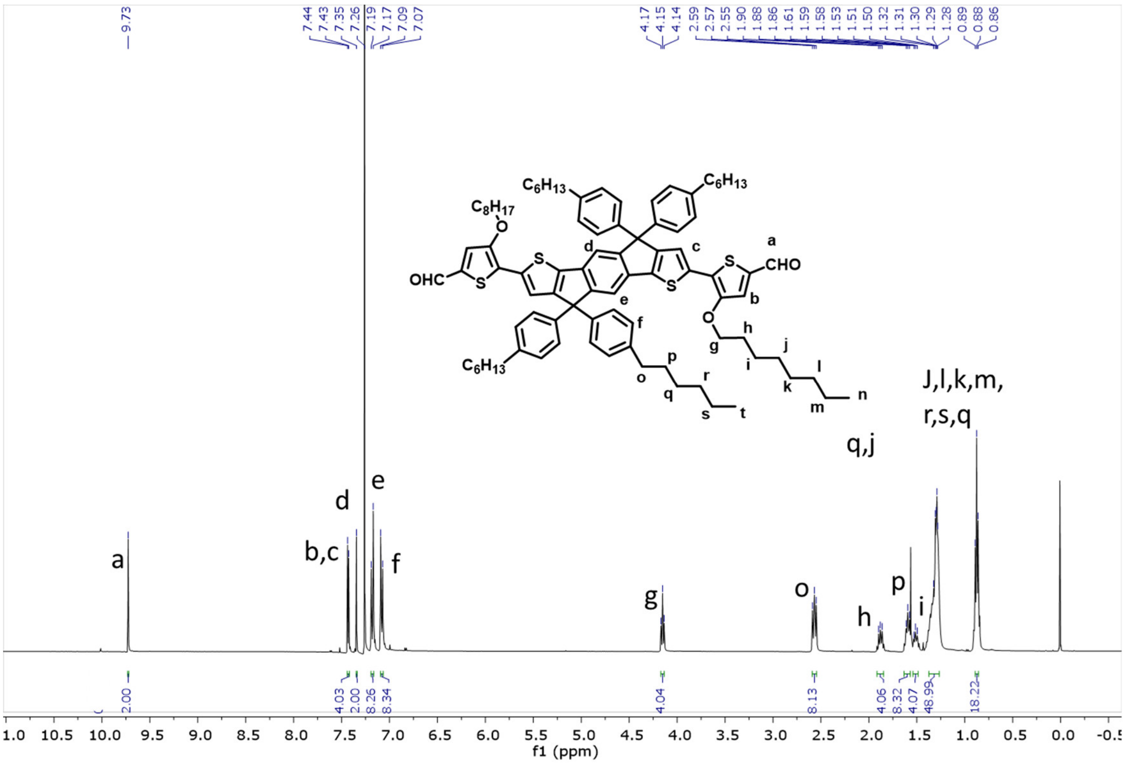

Figure S1. ^{1}H NMR spectrum (400 MHz, $CDCl_3$) of 7a.

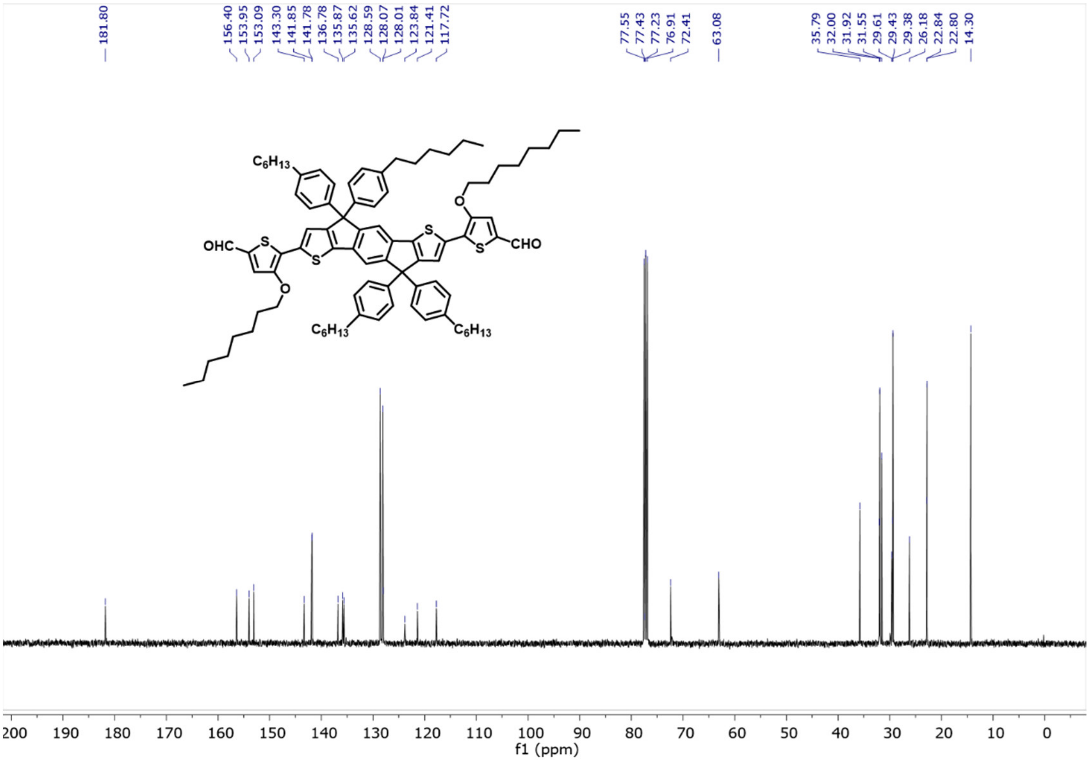

Figure S2. ^{13}C NMR spectrum (400 MHz, $CDCl_3$) of 7a.

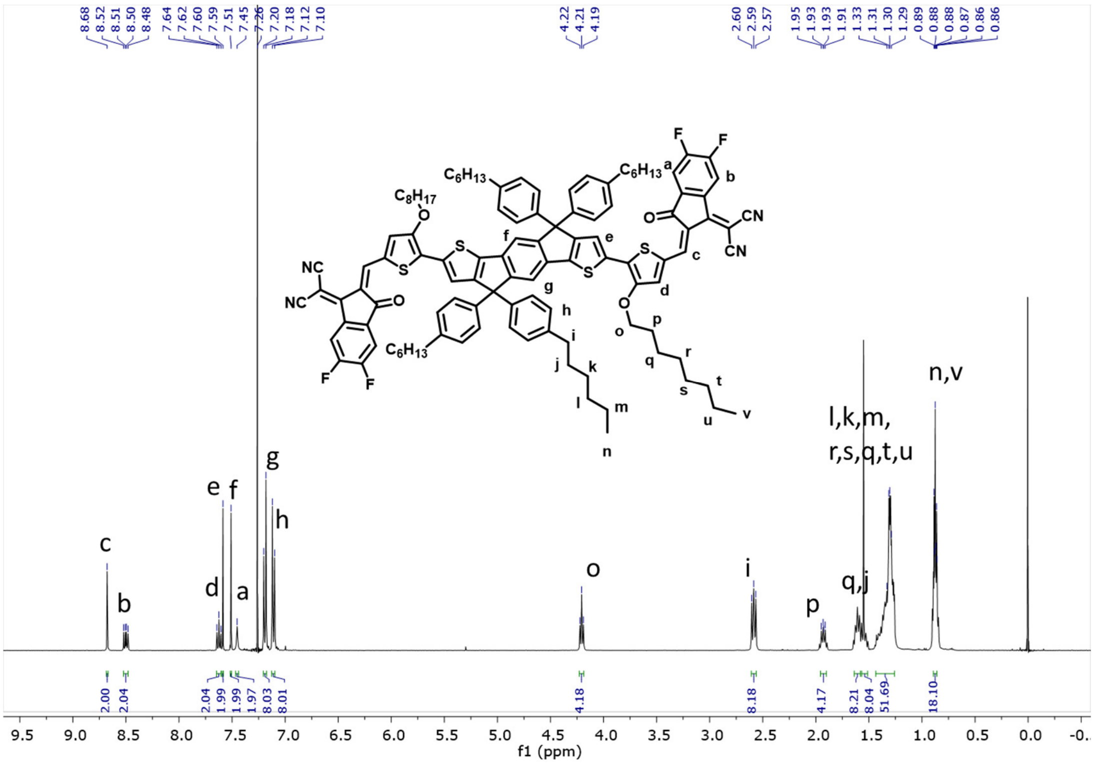

Figure S3. ^{1}H NMR spectrum (400 MHz, $CDCl_3$) of IOICO-4F.

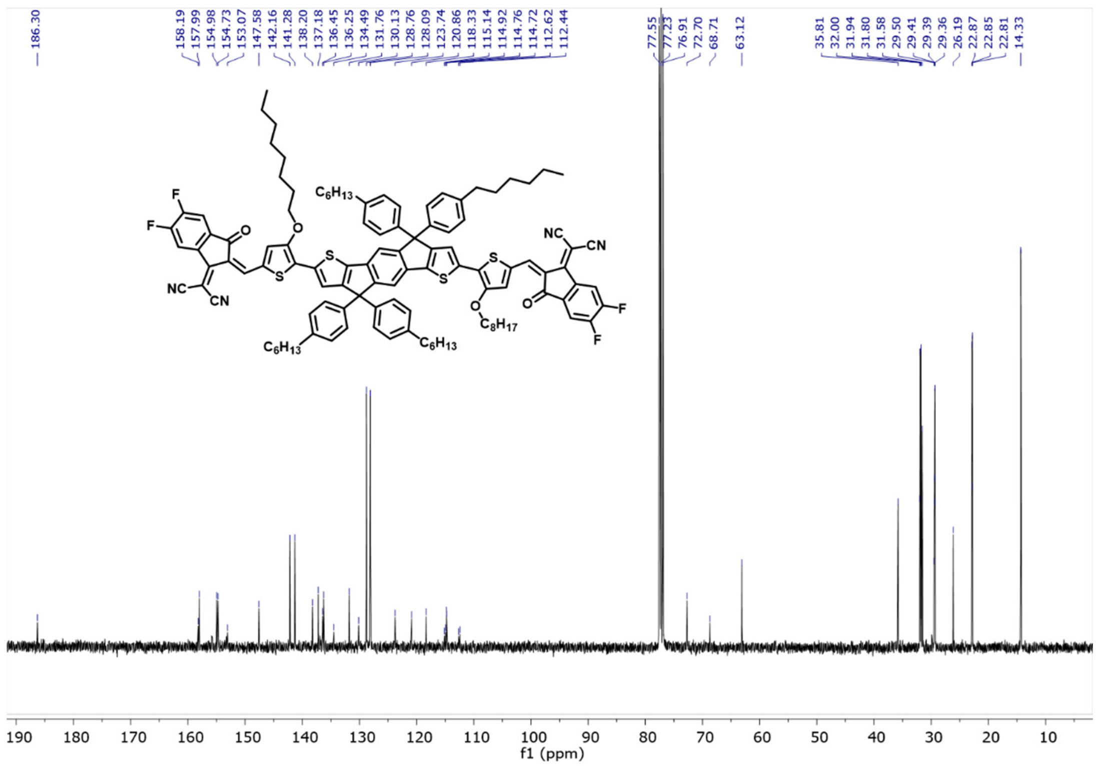

Figure S4. ^{13}C NMR spectrum (400 MHz, $CDCl_3$) of IOICO-4F.

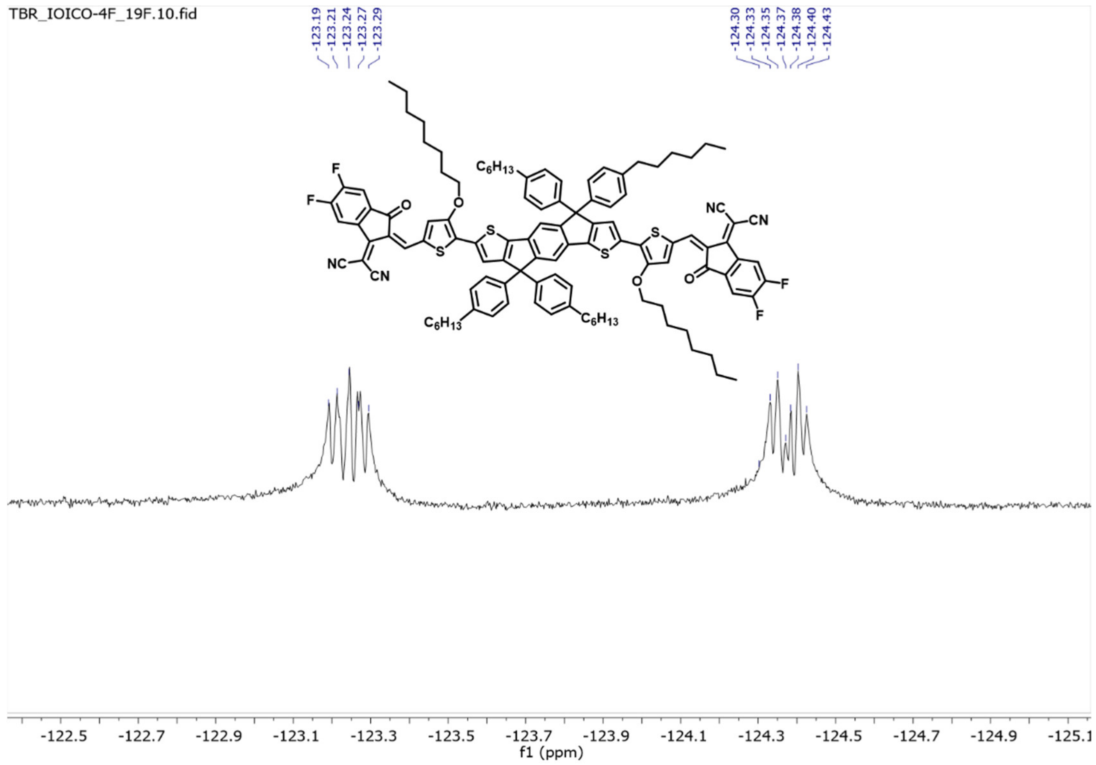

Figure S5. ^{19}F NMR spectrum (400 MHz, $CDCl_3$) of IOICO-4F.

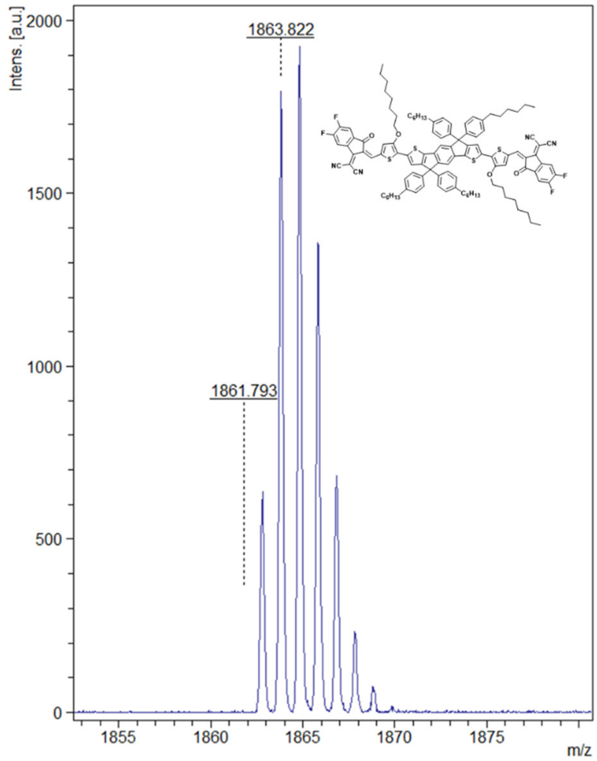

Figure S6. MALDI-TOF mass spectrum of IOICO-4F.

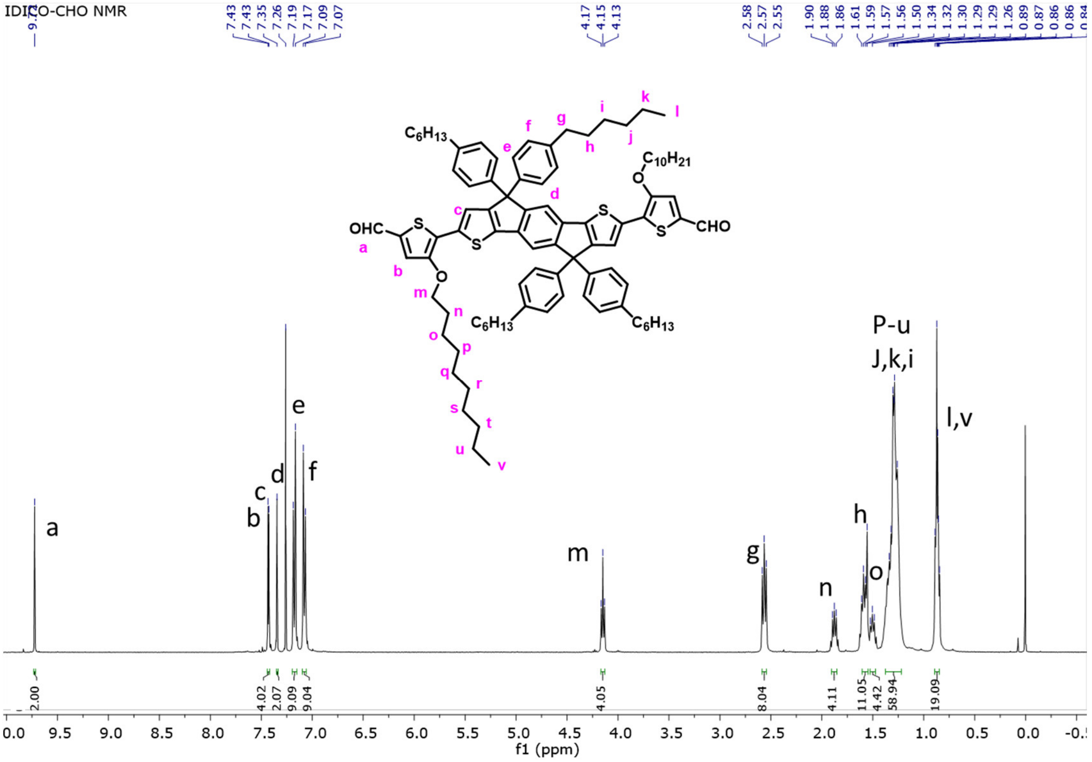

Figure S7. ^{1}H NMR spectrum (400 MHz, $CDCl_3$) of 7b.

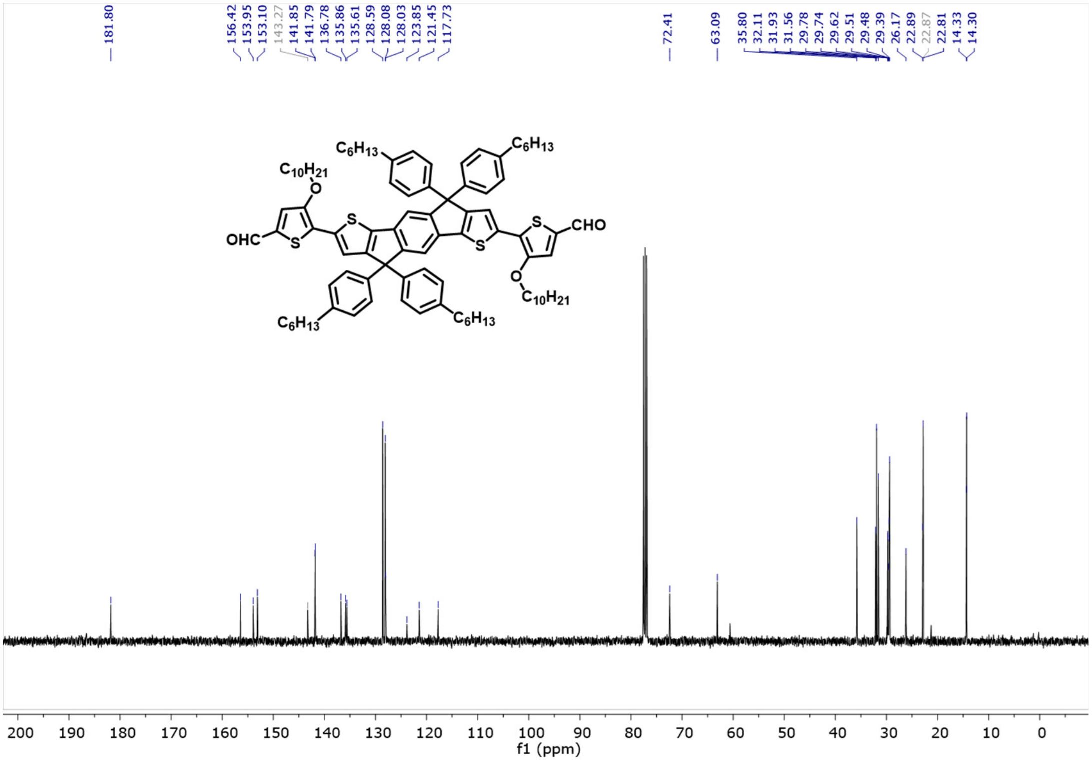

Figure S8. ^{13}C NMR spectrum (400 MHz, $CDCl_3$) of 7b.

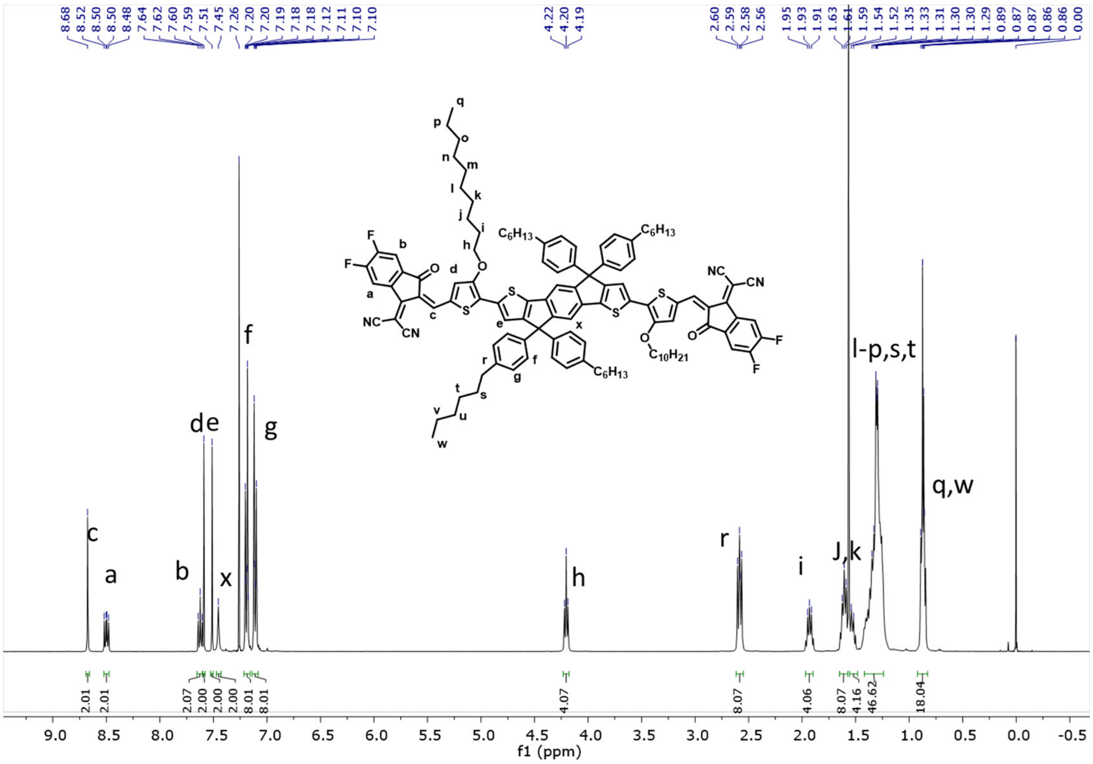

Figure S9. ^{1}H NMR spectrum (400 MHz, $CDCl_3$) of IDICO-4F.

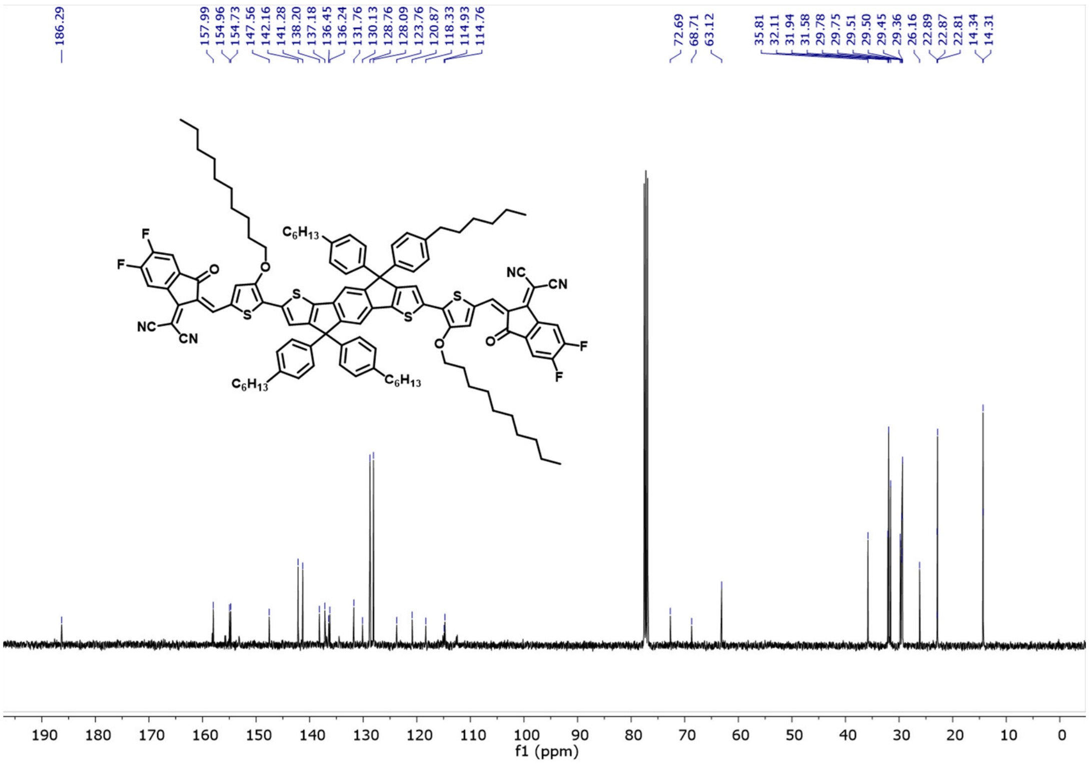

Figure S10. ^{13}C NMR spectrum (400 MHz, $CDCl_3$) of IDICO-4F.

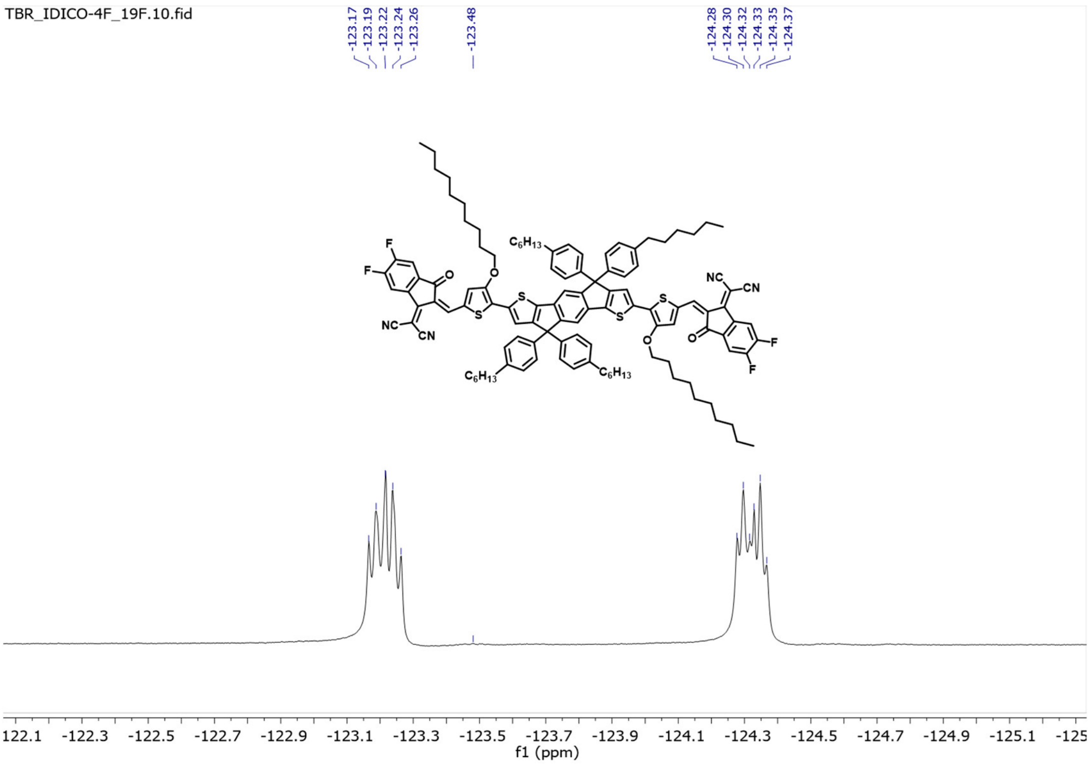

Figure S11. ^{19}F NMR spectrum of IDICO-4F.

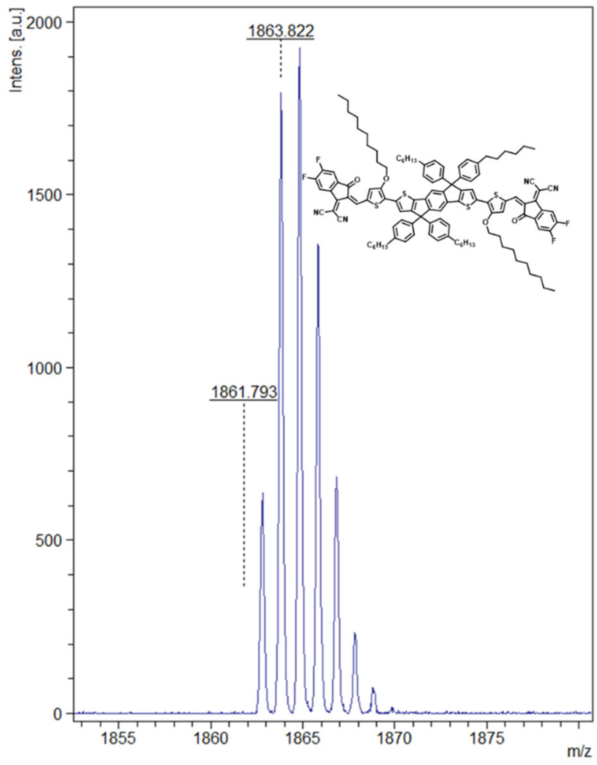

Figure S12. MALDI-TOF mass spectrum of IDICO-4F.

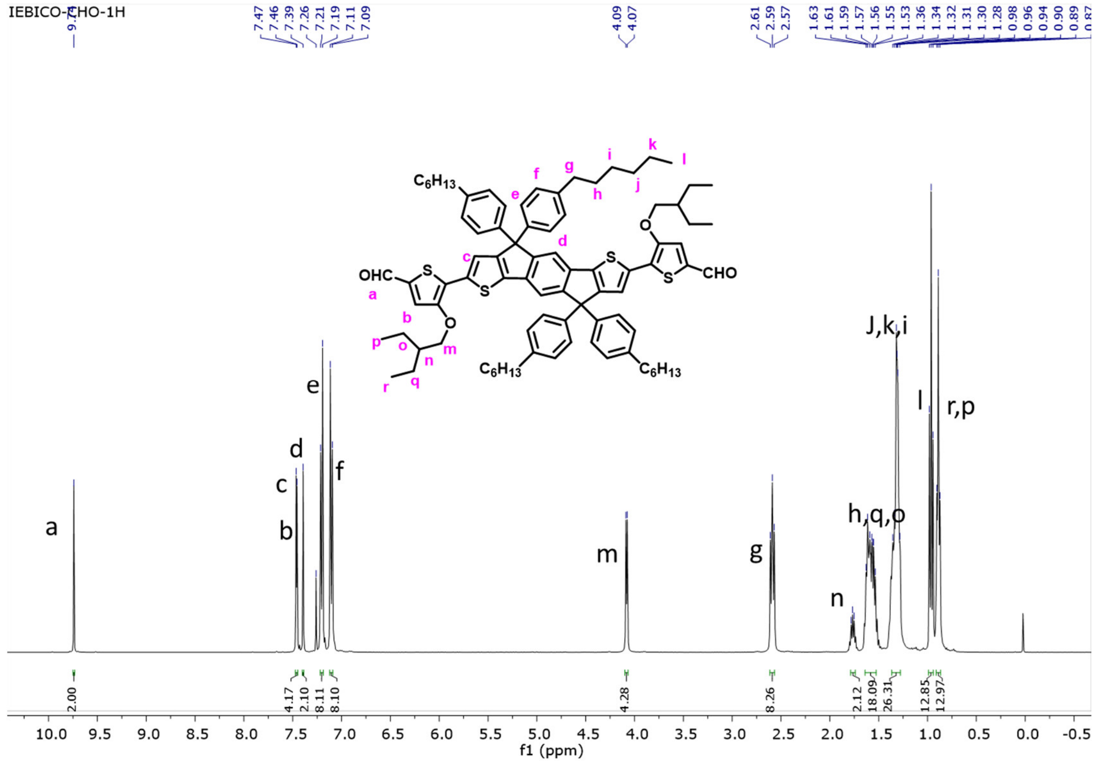

Figure S13. ^{1}H NMR spectrum (400 MHz, $CDCl_3$) of 7c.

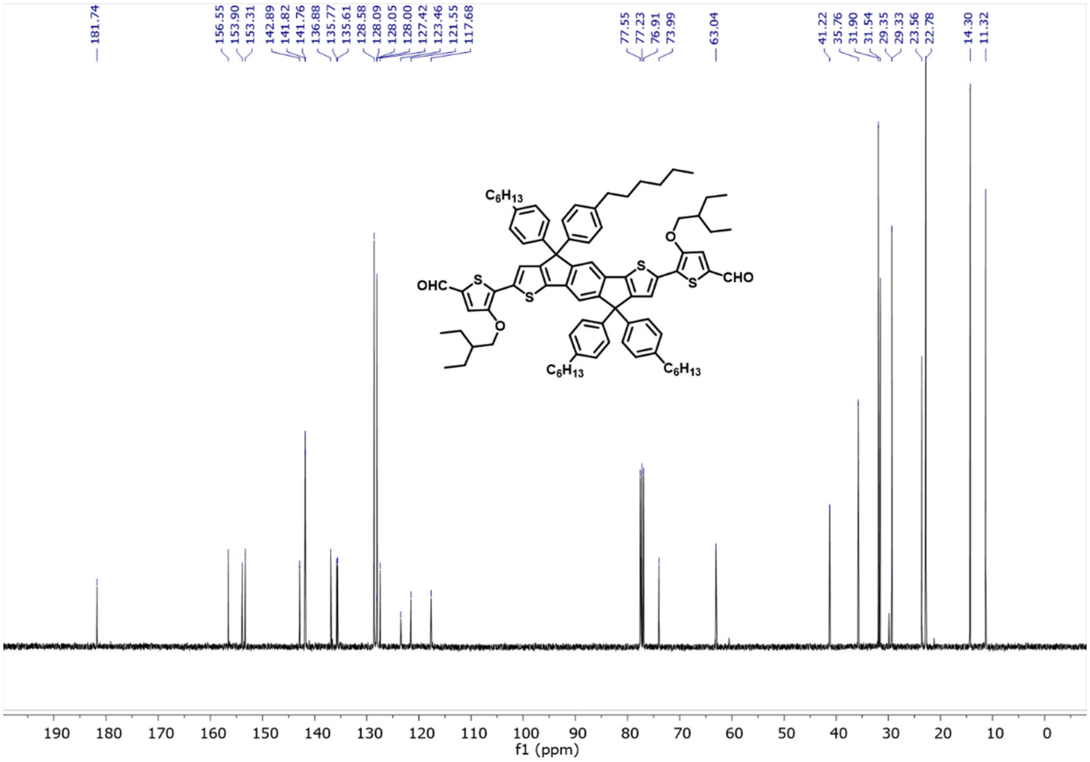

Figure S14. ^{13}C NMR spectrum (400 MHz, $CDCl_3$) of 7c.

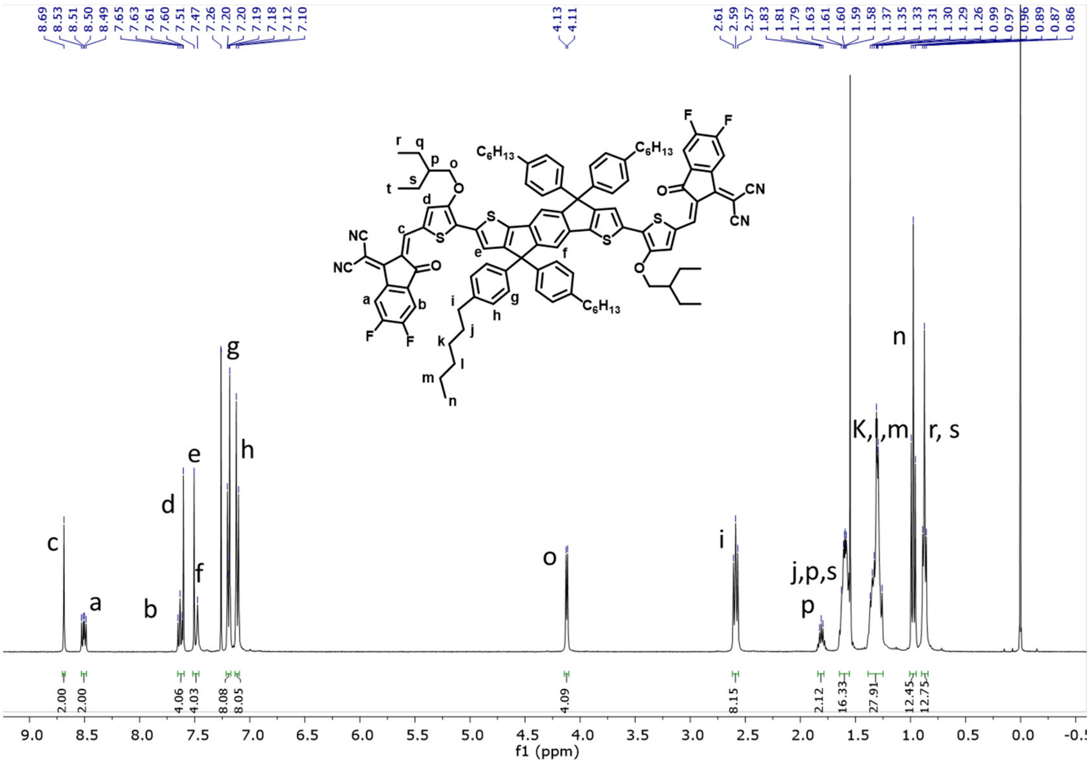

Figure S15. ^{1}H NMR spectrum (400 MHz, $CDCl_3$) of IEBICO-4F.

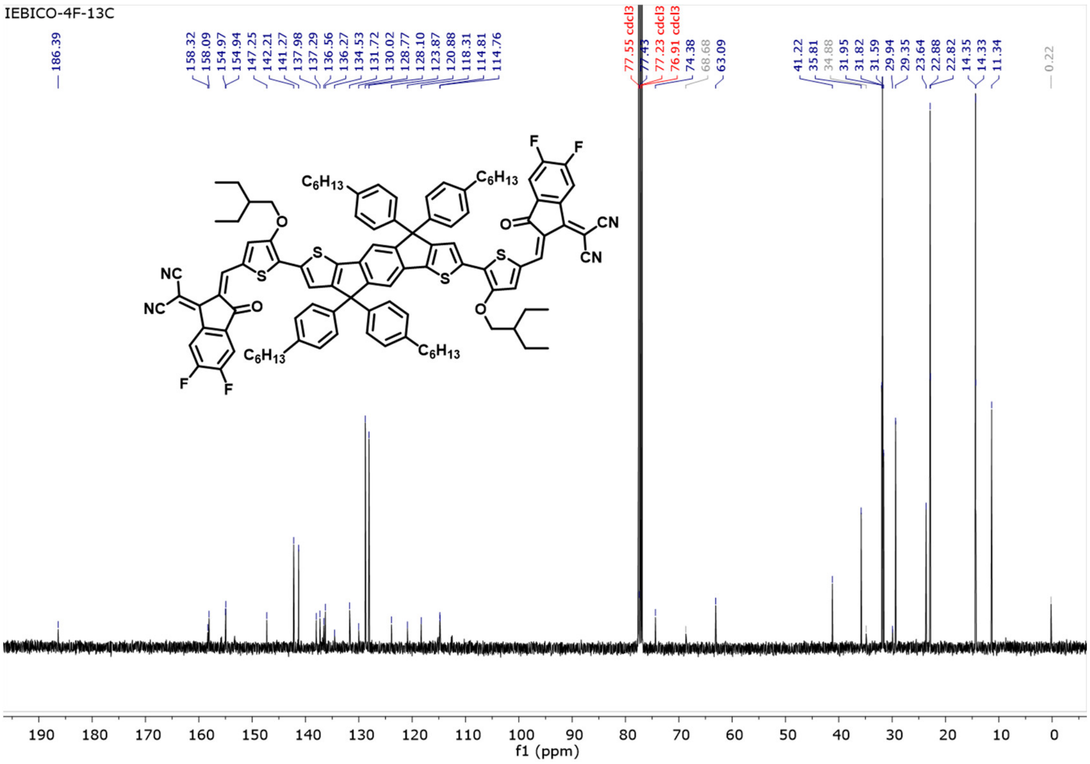

Figure S16. ^{13}C NMR spectrum (400 MHz, $CDCl_3$) of IEBICO-4F.

Device Fabrication and Characterization

All the devices are based on an inverted sandwich structure, the patterned indium-tin oxide glass (ITO glass)/zinc oxide (ZnO) (ca. 30 nm)/PTB7-Th:IRICO-4F/V_2O_5 (ca. 5.5 nm)/Ag (ca. 100 nm). First, ITO glass (sheet resistance = 15 Ω) was continuously pre-cleaned in the ultrasonic bath with de-ionized water, acetone, and isopropanol. Then, a ZnO layer was spin-coated at 4000 r·min^{-1} onto the ITO glass from ZnO precursor solution (prepared by dissolving 0.1 g of zinc acetate dihydrate ($Zn(CH_3COO)_2{\cdot}2H_2O$) and 0.03 mL of ethanolamine ($NH_2CH_2CH_2OH$) in 1 mL of 2-methoxyethanol ($CH_3OCH_2CH_2OH$)), followed by baking at 200 °C for 30 min. The active layer blend (20 mg mL^{-1} in chlorobenzene) was spin-coated at 2500 r·min^{-1} onto the ZnO layer to form the photoactive layer. A V_2O_5 layer and Ag layer were then evaporated under vacuum (ca. 10^{-5} Pa) to form the anode electrode. Cell sizes were 0.12 or 0.2 cm^2. The *J-V* curves was measured by using a computer-controlled source meter (Series 2400, Keithley Instruments, Inc.,USA) and a solar simulator with a 150 W Xenon lamp as a light source equipped with a KG-5 filter (K401, McScience, Inc., Republic of Korea). Its light intensity was adjusted to AM 1.5 G 1 sun light intensity (100 mW/ cm^2) using a calibrated mono Si solar cell. The external quantum efficiency (EQE) spectra were measured in the region about 300 to 1000 nm of the wavelength range, using Incident Photon Conversion Efficiency test system (K3100 IQX,

McScience, Inc., Republic of Korea) which are equipped with a 300 W xenon arc lamp, a monochromator, and an optical chopper (MC 2000, Thorlabs, Inc., USA).

Electrochemical Impedance Spectroscopy measurements

For EIS measurement of PTB7-Th:IRICO-4F, the device structure was prepared as follows: ITO/PEDOT:PSS/Active layer/Al. The measurements were performed in the frequency range from 1×10^3 to 1×10^5 Hz. From the obtained capacitance of blend films at different frequencies, the relative permittivity(ε_r) were calculated from the following equation:

$$\varepsilon_r = \frac{Cd}{\varepsilon_0 A}$$

where C is the capacitance, d is the thickness of the active layer, ε_0 is the permittivity of free space, and A is the device area. Calculated relative permittivity values were 3.83, 4.11, 4.17, and 3.50 for PTB7-Th:IEBICO-4F, PTB7-Th:IEHICO-4F, PTB7-Th:IOICO-4F, and PTB7-Th:IDICO-4F blend films, respectively (see Figure S19). EIS measurements were obtained from using a Potentiostat (AMETEK VersaSTAT4).

SCLC Measurements

Hole-only or electron-only devices were fabricated as follows: ITO/PEDOT:PSS/PTB7-Th:IRICO-4F/MoO_3/Al for holes and ITO/ZnO/Phen-NaDPO/PTB7-Th:IRICO-4F/Phen-NaDPO/Ag for electrons. The mobility was extracted by fitting the *J-V* curves using space charge limited current (SCLC) method, which follows:

$$J = (9/8)\mu\varepsilon_r\varepsilon_0 V^2/d^3.$$

Here, J refers to the current density, μ is hole or electron mobility, ε_r is relative permittivity of the transport medium, ε_0 is the permittivity of free space (8.85×10^{-12} F m^{-1}), $V = V_{appl} - V_{bi}$, where V_{appl} is the applied voltage to the device, and V_{bi} is the built-in voltage due to the difference in work function of the two electrodes (for hole-only diodes, V_{bi} =0 V; for electron-only diodes, V_{bi} is 0.2 V). d is the thickness of the active layer.

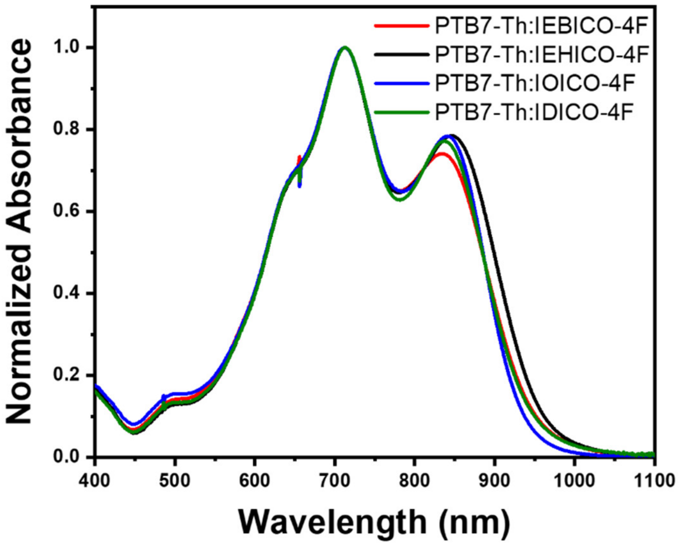

Figure S17. UV-Visible spectra of PTB7-Th:IRICO-4F blend films.

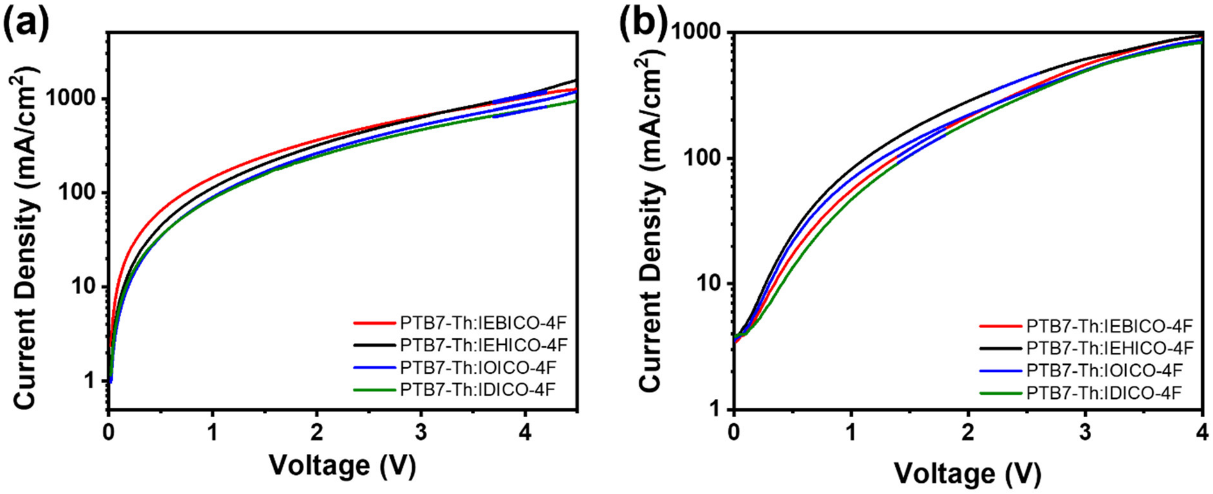

Figure S18. *J-V* characteristics in the dark for (a) hole-only and (b) electron-only devices based on PTB7-Th:IRICO-4F blends.

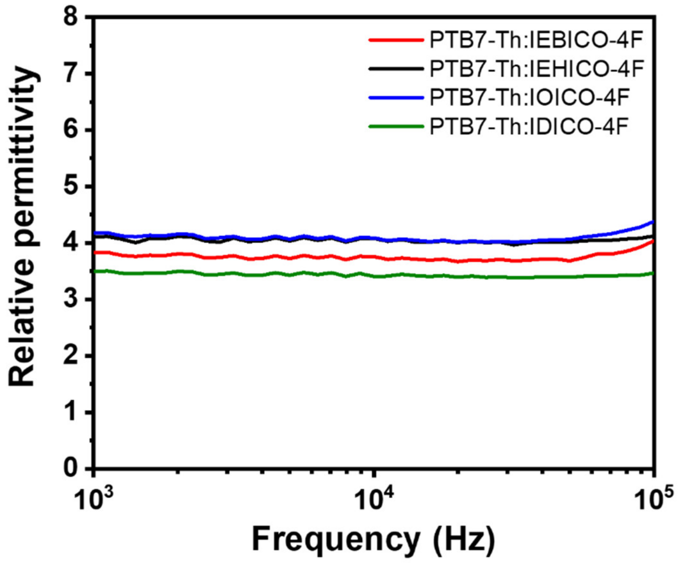

Figure S19. The relative permittivity of PTB7-Th:IRICO-4F blend films in the range of $10^3 – 10^5$ Hz.

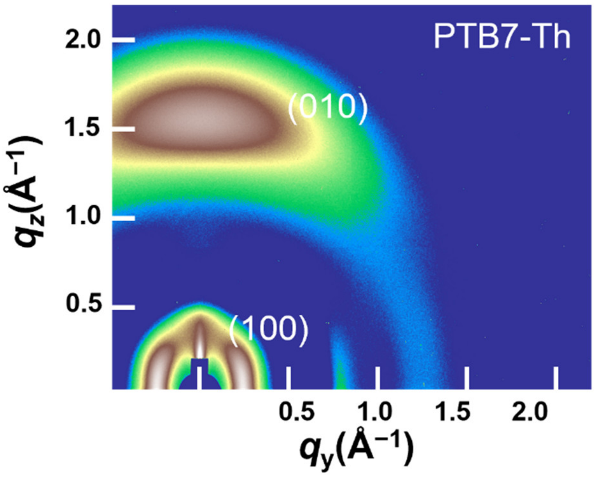

Figure S20. GIWAXS image of PTB7-Th film.

Table S1. The solubilities of the IEBICO-4F, IEHICO-4F, IOICO-4F, and IDICO-4F for tetrahydrofuran, chloroform, and chlorobenzene at room temperature.

NFAs	Tetrahydrofuran	Chloroform	Chlorobenzene
IEBICO-4F	7 mg/mL	21 mg/mL	25 mg/mL
IEHICO-4F	8 mg/mL	22 mg/mL	30 mg/mL
IOICO-4F	11 mg/mL	60 mg/mL	20 mg/mL
IDICO-4F	6 mg/mL	90 mg/mL	33 mg/mL

Table S2. Charge Mobilities of blend films measured by SCLC method. Each hole (h^+) mobilities and electron (e^-) mobilities are averaged for 8 devices.

Active layer	h^+ mobility ($cm^2/(V\cdot s)$)	e^- mobility ($cm^2/(V\cdot s)$)	h^+/e^- ratio
PTB7-Th:IEBICO-4F	$1.11(\pm0.28)\times10^{-4}$	$1.01(\pm0.04)\times10^{-4}$	1.10
PTB7-Th:IEHICO-4F	$1.23(\pm0.09)\times10^{-4}$	$1.30(\pm0.08)\times10^{-4}$	0.95
PTB7-Th:IOICO-4F	$9.38(\pm1.8)\times10^{-5}$	$9.58(\pm0.23)\times10^{-5}$	0.98
PTB7-Th:IDICO-4F	$1.00(\pm0.26)\times10^{-4}$	$9.40(\pm0.50)\times10^{-5}$	1.06
